# Novel machine-learning analysis of SARS-CoV-2 infection in a subclinical nonhuman primate model using radiomics and blood biomarkers

**DOI:** 10.1038/s41598-023-46694-9

**Published:** 2023-11-10

**Authors:** Winston T. Chu, Marcelo A. Castro, Syed Reza, Timothy K. Cooper, Sean Bartlinski, Dara Bradley, Scott M. Anthony, Gabriella Worwa, Courtney L. Finch, Jens H. Kuhn, Ian Crozier, Jeffrey Solomon

**Affiliations:** 1 Center for Infectious Disease Imaging, Radiology and Imaging Sciences, Clinical Center, National Institutes of Health, Bethesda, MD USA; 2 Integrated Research Facility at Fort Detrick, Division of Clinical Research, National Institute of Allergy and Infectious Diseases, National Institutes of Health, Frederick, MD USA; 3 Clinical Monitoring Research Program Directorate, Frederick National Laboratory for Cancer Research, Frederick, MD USA

**Keywords:** Machine learning, Diagnostic markers, Viral infection, Image processing

## Abstract

Detection of the physiological response to severe acute respiratory syndrome coronavirus 2 (SARS-CoV-2) infection is challenging in the absence of overt clinical signs but remains necessary to understand a full subclinical disease spectrum. In this study, our objective was to use radiomics (from computed tomography images) and blood biomarkers to predict SARS-CoV-2 infection in a nonhuman primate model (NHP) with inapparent clinical disease. To accomplish this aim, we built machine-learning models to predict SARS-CoV-2 infection in a NHP model of subclinical disease using baseline-normalized radiomic and blood sample analyses data from SARS-CoV-2-exposed and control (mock-exposed) crab-eating macaques. We applied a novel adaptation of the minimum redundancy maximum relevance (mRMR) feature-selection technique, called mRMR-permute, for statistically-thresholded and unbiased feature selection. Through performance comparison of eight machine-learning models trained on 14 feature sets, we demonstrated that a logistic regression model trained on the mRMR-permute feature set can predict SARS-CoV-2 infection with very high accuracy. Eighty-nine percent of mRMR-permute selected features had strong and significant class effects. Through this work, we identified a key set of radiomic and blood biomarkers that can be used to predict infection status even in the absence of clinical signs. Furthermore, we proposed and demonstrated the utility of a novel feature-selection technique called mRMR-permute. This work lays the foundation for the prediction and classification of SARS-CoV-2 disease severity.

## Introduction

Severe acute respiratory syndrome coronavirus 2 (SARS-CoV-2) has caused over 771 million confirmed cases of coronavirus disease 2019 (COVID-19) and over 6 million reported deaths worldwide as of December 21st, 2022^1^. As the pandemic has progressed, so has the urgent need for diagnostic and prognostic systems to quickly and accurately predict exposure, infection, and disease states to enable timely medical intervention and improve patient outcomes. Although considerable progress has been made to provide accurate diagnostic testing for “infection” status, capturing the “disease” spectrum (response to infection) has been more challenging, particularly as up to one-half of all SARS-CoV-2-infected individuals, even more among young and healthy populations, are paucisymptomatic or asymptomatic^2–7^.

Historically, computed tomography (CT) has been applied to diagnose viral pneumonia; indeed, recent research suggests the utility of lung CT to support the diagnosis of severe SARS-CoV-2 infection and COVID-19. In some settings, CT scanning improved diagnostic sensitivity and false-negative rates compared to reverse transcription-polymerase chain reaction (RT-PCR)-based detection of SARS-CoV-2 nucleic acids^8–11^. The degree of lung involvement detected via CT is positively correlated with clinical severity, viral load, and outcome in adults and children^12–16^. Of note, CT imaging of patients with paucisymptomatic or asymptomatic disease has revealed lung abnormalities not apparent by routine clinical evaluation^17^.

Many studies have characterized human SARS-CoV-2 infection and COVID-19^18–21^. However, understanding the relationship between infection and disease is challenged in clinical settings by several factors: pre-exposure baseline measurements are usually unavailable; data from the immediate post-exposure/infection period prior to presentation as a COVID-19 case is scant, and properly accounting for dose/route of exposure, time from exposure, medical history, immune status, and other environmental factors is nearly impossible. Addressing these considerations necessitates the development of animal models of SARS-CoV-2 exposure, infection, and disease for studies that include robust controls of environmental factors, a standardized baseline, and serial longitudinal evaluation.

Although experimental SARS-CoV-2 exposure of nonhuman primates (NHPs) has thus far not yielded severe disease, mild and/or subclinical disease, seen in the majority of the human population, is accurately modeled in NHPs of a variety of species^22,23^. Furthermore, CT imaging of SARS-CoV-2-infected NHPs reveals otherwise clinically “silent” lung abnormalities, that are very characteristic of COVID-19^24,25^. These tomographic footprints of disease can be quantified to distinguish between infected and uninfected animals and to track the progression of pulmonary disease longitudinally. However, even with advanced quantification techniques, vast amounts of radiomic data (calculated from CT images) from these experiments remain unexplored. Independent of, or in conjunction with clinical pathological and immunological features, analysis of radiomic data may further enhance the understanding of and improve quantifiable performance benchmarks for therapies targeting the asymptomatic to paucisymptomatic COVID-19 spectrum.

Radiomic, clinical pathological, and immunological analyses measure a wide range of physiologic and pathophysiologic data, but only a subset is likely to be altered by SARS-CoV-2 infection and subsequent disease states. Choosing meaningful data subsets (“feature selection”) therefore is crucial to remove irrelevant or redundant data that impairs model performance. Feature selection is the process of reducing the number of input variables to improve the generalizability, interpretability, computational cost, and performance of a particular model. Feature selection is particularly important for studies with many dependent variables but relatively few samples due to the “curse of dimensionality” (i.e., when the number of samples is insufficient to accurately estimate model parameters) and must be driven by subject-matter expertise in addition to data-driven approaches^26^. However, many data-driven feature-selection techniques require users to choose a score or size threshold that is not based on statistics, introducing a potential source of bias and increasing the likelihood of overfitting (i.e., modeling the original data set too closely, reducing generalizability). Minimum redundancy maximum relevance (mRMR) is one such example of a data-driven feature-selection approach that is becoming increasingly popular due to its performance and low computation cost but it also requires users to choose a size threshold for the feature set^27^. This motivated our team to develop an adaptation of mRMR, called “mRMR-permute”, that uses permutation testing to choose an mRMR score threshold based on a statistical threshold (i.e., a *p* value).

In this study, we aggregated CT imaging, clinical pathological, and immunological data to build a predictive model to differentiate SARS-CoV-2-infected from uninfected control crab-eating macaques. To accomplish this goal, we obtained a rich dataset with pre-exposure baseline measurements of 170 (111 radiomic, 37 clinical pathological, and 22 immunological) features. Using data normalized to each macaque’s pre-exposure values, we performed domain-specific feature screening and applied a thorough array of data-driven feature-selection techniques to identify the top-performing feature set. In addition to 9 popular data-driven feature-selection techniques (3 f.-test *p* value thresholds, 2 logistic regression-based, 3 tree-based, and mRMR), we also applied mRMR-permute for statistically-thresholded and unbiased feature selection. The features selected for our top-performing model inform further development of predictive models targeting disease severity and outcome in infected macaques.

## Results

### Demographics and clinical assessment data

Across both the virus-infected and control groups, the average age was 5.0 years, and the average weight was 4.3 kg at baseline. There was no significant difference between groups (*p* > 0.05) in age, sex, country of origin, inoculation mode, weight, or average change in weight. In addition, there was no difference in maximum clinical score between groups—demonstrating that the groups were indistinguishable based on clinical presentation alone (Table 1). Of note, the clinical scores were relative to a maximum score of 40, without any animals exceeding a score of 5.

**Table 1:** Cohort description.

	SARS-CoV-2	Mock	Statistic (df); *p* value
N [animals (samples)]	12 (48)	8 (32)	
Sex (M/F)	6/6	4/4	X^2^ (1) = 0; *p* = 1
Country of origin (Cambodia/Indonesia)	6/6	4/4	X^2^ (1) = 0; *p* = 1
Intrabronchial/Aerosol	9/3	6/2	X^2^ (1) = 0; *p* = 1
Age (yr)	5.2 ± 1.2	4.8 ± 1.0	Welch’s t (17) = 0.76; *p* = 0.46
Baseline weight (kg)	4.17 ± 1.11	4.49 ± 1.65	Welch’s t (11) = − 0.48; *p* = 0.64
Average change in weight (kg)	− 0.03 ± 0.11	− 0.04 ± 0.14	Welch’s t (13) = 0.25; *p* = 0.81
Maximum post-infection clinical score	3.4 ± 0.6	2.6 ± 1.4	Welch’s t (9) = 1.61; *p* = 0.14

*M/F* male/female; *SD* standard deviation; *SARS-CoV-2* severe acute respiratory syndrome coronavirus 2; *df* degree of freedom.

Mean ± standard deviation and counts are given for quantitative and categorical descriptors, respectively.

Clinical scoring is on a 0-40 scale.

Welch’s *t*-test was used to test for between-group differences and the resultant test statistics and *p* values are given.

### Feature correlations

**Figure 1:**
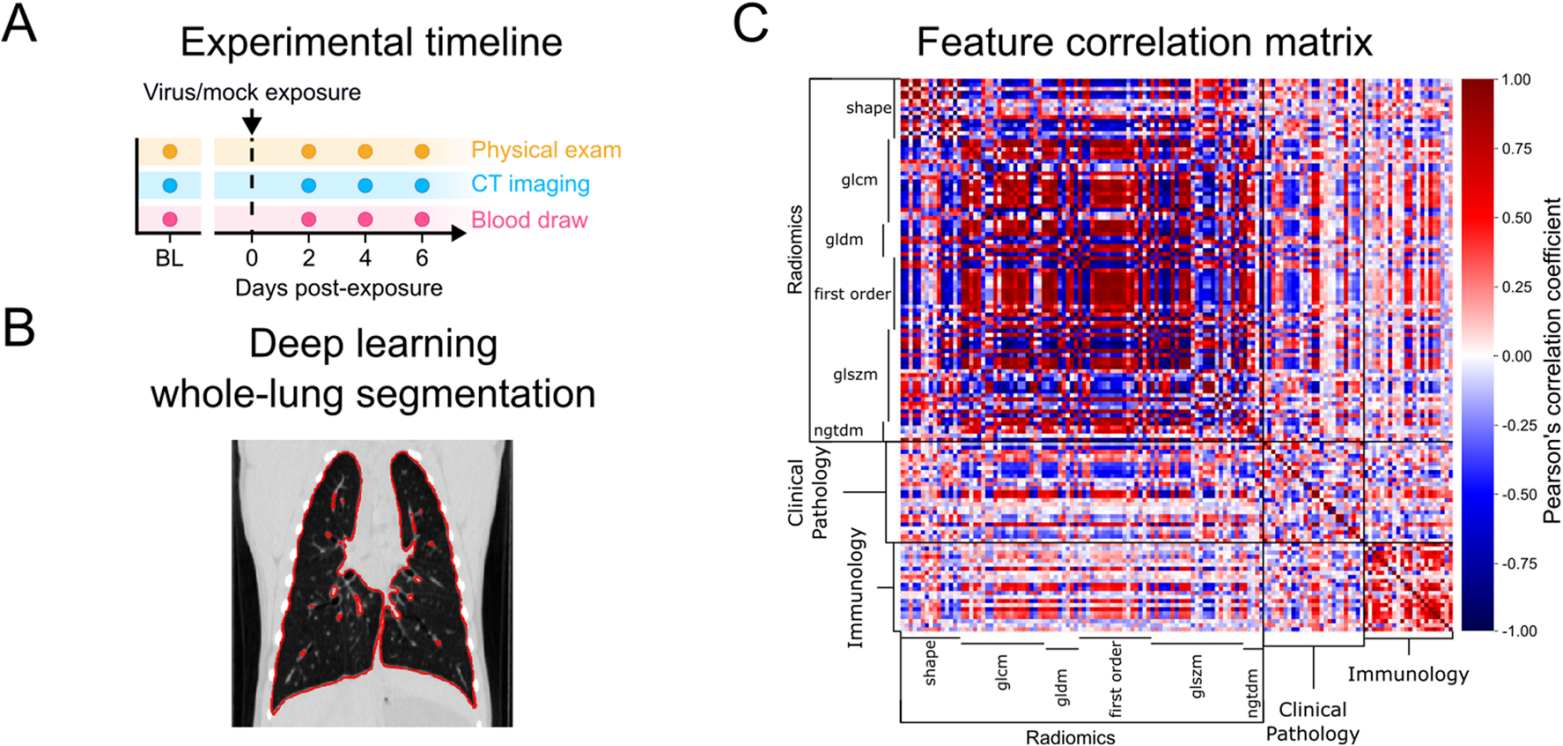
Study methods and feature characterization. (**A**) Experimental timeline: Macaques were either SARS-CoV-2 exposed or mock-exposed. All animals underwent physical examination, CT imaging, and blood draws pre-exposure, 2-, 4-, and 6-days post-exposure. (**B**) Deep learning whole lung segmentation: A previously described deep-learning model trained on radiologist-labeled CT scans of crab-eating macaques was applied to produce whole-lung segmentations for this study. (**C**) Feature correlation matrix for 4 days change from baseline: Correlations between radiomic, clinical pathological, and immunological features were calculated. The high number of highly correlated features illustrates the need for dimensionality reduction. SARS-CoV-2—severe acute respiratory syndrome coronavirus 2; CT—computed tomography; glcm—gray-level co-occurrence matrix; gldm—gray-level dependence matrix; glszm—gray-level size zone matrix; ngtdm—neighboring gray-tone difference matrix.

An exploratory correlation analysis was conducted between features to assess macro trends in the data and determine if dimensionality reduction was necessary. Figure 1 contains the generated correlation matrix. The average absolute magnitude of Pearson’s correlation coefficient (|r|) resultant from comparison of all radiomic, clinical pathological, and immunological features was 0.36 ± 0.28 (mean ± standard deviation [SD]). Of the 9,316 correlations, 1,545 (16%) had a value equal to or greater than 0.7, classifying them as strong to very strong^28^. The average |r| for intra-domain correlations of radiomic, clinical pathological, and immunological features were 0.53 ± 0.29, 0.24 ± 0.19, and 0.34 ± 0.25 (mean ± SD), respectively. These corresponded with 36, 2, and 11% of correlations, respectively, being strong to very strong^28^. The average |r| for inter-domain correlations between radiomic and clinical pathological, radiomic and immunological, and clinical pathological and immunological features were 0.26 ± 0.19, 0.21 ± 0.17, and 0.20 ± 0.16 (mean ± SD), respectively. These corresponded with 3, 1, and 0% of correlations, respectively, being strong to very strong^28^. The absolute magnitudes of correlations between features, particularly intra-domain, indicate a high likelihood of redundant features and motivated the application of dimensionality reduction techniques, such as domain-specific feature screening and data-driven feature selection.

### Model development

#### Domain-specific feature screening and data-driven feature selection

**Figure 2:**
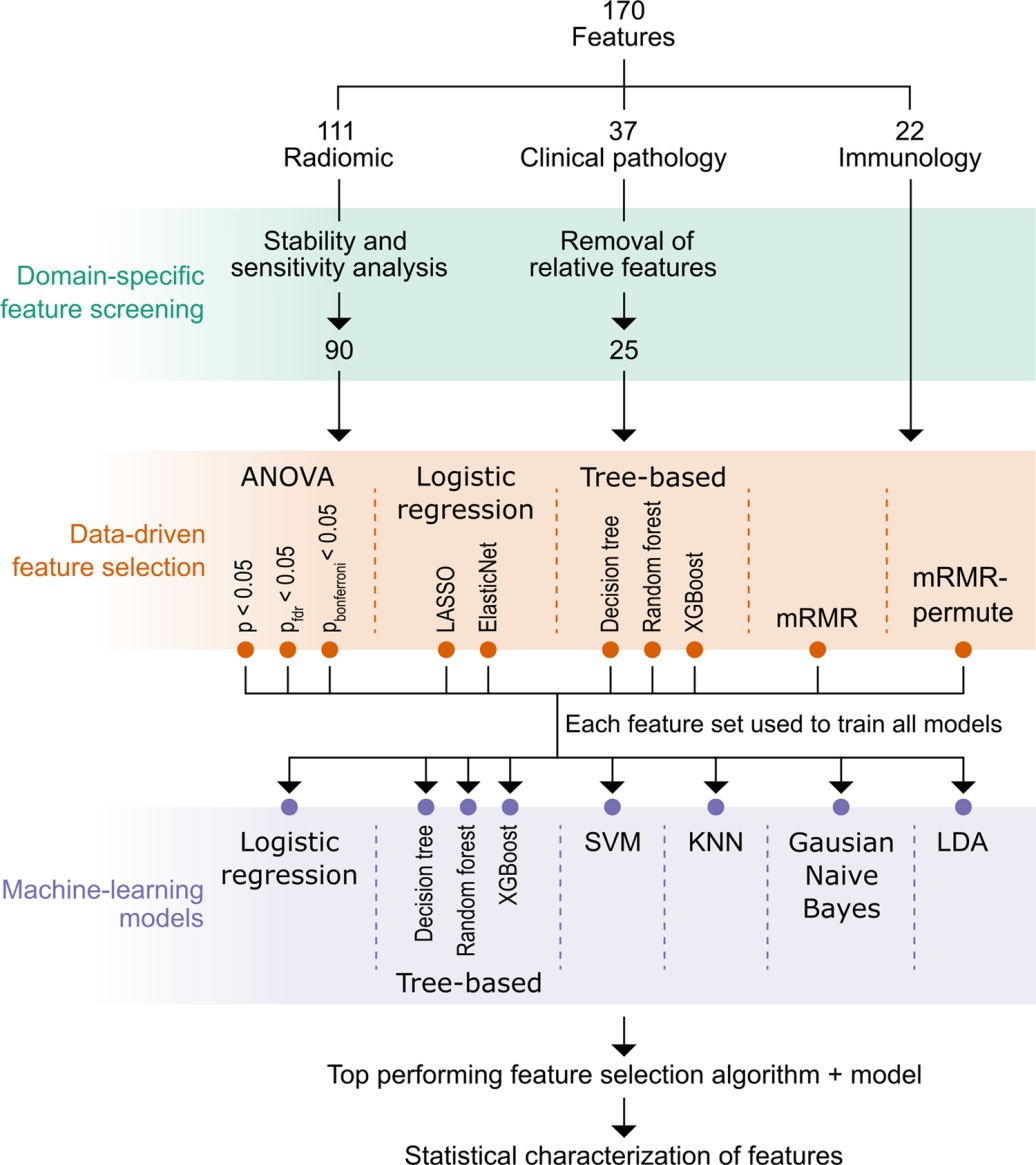
Dimensionality reduction. A total of 170 features were derived from CT images and blood draws. Domain-specific feature screening was used to reduce the feature space based on recommendations from subject-matter experts. This was followed by data-driven feature selection. 10 feature selection algorithms were tested by using each feature set to train 8 popular machine-learning models. The best-performing feature set and model were identified and further characterized. ANOVA—analysis of variance; *p*_fdr_—*p* value corrected for the false-discovery rate; *p*_bonferroni_—*p* value after Bonferroni correction for multiple comparisons; mRMR—minimum redundancy maximum relevance; SVM—support vector machine; KNN—k-nearest neighbors; LDA—linear discriminant analysis.

Out of 111 radiomic features extracted from scans using PyRadiomics, 90 passed exclusion criteria defined by a stability and sensitivity analysis^29^. By recommendation of a board-certified diplomate in anatomic pathology (co-author T.K.C.), 12 clinical pathological features were excluded because they were either derived from other features or deemed not to contribute meaningfully to the predictive determination. No immunological features were recommended for exclusion by an immunologist due to the lack of redundancy within the examined features (co-author S.M.A.). The 137 remaining radiomic, clinical pathological, and immunological features were further reduced using an array of data-driven techniques (Fig. 2).

Ten data-driven feature-selection techniques were examined in this study. Supplemental Table 1 shows the distribution of features across the 3 domains for each feature set. Mixed-effects analyses of variance (ANOVAs) were used to measure the effect of class and time on each feature. Class-effect *p* value thresholds of *p* < 0.05, *p* value corrected for the false-discovery rate using the Benjamini–Hochberg correction (*p*_fdr_) < 0.05, and p value after Bonferroni correction for multiple comparisons (*p*_bonferroni_) < 0.05 were used to select features and resulted in features sets of size 71, 49, and 8, respectively. Supplemental Table 2 shows the results of the mixed-effects ANOVAs, performed on all features. Regression-based feature-selection techniques, using least absolute shrinkage and selection operator (LASSO) and ElasticNet scores (score threshold of 0), resulted in 10 and 74 features, respectively. Tree-based feature-selection techniques, using feature importance scores from decision tree, random forest, and XGBoost (importance score threshold of 0), resulted in 3, 48, and 13 features, respectively.

**Table 2:** Class effect on mRMR-permute features.

Rank	Feature	Modality	Class effect			Time effect		Class–time interaction effect	
			Direction	η_p_^2^	*p*_fdr_	η_p_^2^	*p*_fdr_	η_p_^2^	*p*_fdr_
1	Uniformity-first order	Radiomics	SARS-CoV-2 < Control	0.64	7.29E − 04	0.24	3.80E − 02	0.01	9.80E − 01
2	Blood urea nitrogen (mg/dL)	Clinical Pathology	SARS-CoV-2 < Control	0.44	2.83E − 03	0.18	6.42E − 02	0.03	9.80E − 01
3	Busyness-ngtdm	Radiomics	SARS-CoV-2 < Control	0.60	7.29E − 04	0.10	2.02E − 01	0.02	9.80E − 01
4	Spherical disproportion-shape	Radiomics	SARS-CoV-2 > Control	0.25	3.09E − 02	0.14	1.20E − 01	0.06	9.80E − 01
5	Absolute basophil (K/µL)	Clinical Pathology	SARS-CoV-2 < Control	0.33	1.21E − 02	0.16	8.93E − 02	0.29	6.91E − 02
6	Gray level variance-glszm	Radiomics		0.10	1.73E − 01	0.07	3.37E − 01	0.07	9.80E − 01
7	Zone variance-glszm	Radiomics	SARS-CoV-2 < Control	0.58	7.29E − 04	0.10	1.91E − 01	0.03	9.80E − 01
8	Contrast-ngtdm	Radiomics	SARS-CoV-2 < Control	0.40	5.35E − 03	0.03	5.46E − 01	0.01	9.80E − 01
9	Gray level non-uniformity normalized-glrlm	Radiomics	SARS-CoV-2 < Control	0.54	1.25E − 03	0.18	6.42E − 02	0.02	9.80E − 01
10	Maximum 3D diameter-shape	Radiomics	SARS-CoV-2 < Control	0.27	2.72E − 02	0.39	1.80E − 03	0.03	9.80E − 01
11	Median – first order	Radiomics	SARS-CoV-2 > Control	0.47	2.38E − 03	0.41	1.28E − 03	0.06	9.80E − 01
12	Absolute reticulocyte (K/µL)	Clinical Pathology	SARS-CoV-2 < Control	0.24	3.26E − 02	0.03	5.46E − 01	0.10	9.80E − 01
13	Large area emphasis-glszm	Radiomics	SARS-CoV-2 < Control	0.58	7.29E − 04	0.10	1.91E − 01	0.03	9.80E − 01
14	Entropy – first order	Radiomics	SARS-CoV-2 > Control	0.53	1.25E − 03	0.18	6.42E − 02	0.02	9.80E − 01
15	Mesh volume-shape	Radiomics	SARS-CoV-2 < Control	0.48	2.28E − 03	0.23	3.80E − 02	0.01	9.80E − 01
16	Joint energy-glcm	Radiomics	SARS-CoV-2 < Control	0.45	2.83E − 03	0.25	3.80E − 02	0.00	9.80E − 01
17	Sum entropy-glcm	Radiomics	SARS-CoV-2 > Control	0.50	2.02E − 03	0.19	6.42E − 02	0.02	9.80E − 01
18	Joint average-glcm	Radiomics	SARS-CoV-2 > Control	0.45	2.83E − 03	0.22	3.80E − 02	0.00	9.80E − 01
19	Compactness 1-shape	Radiomics	SARS-CoV-2 < Control	0.26	2.88E − 02	0.16	8.93E − 02	0.06	9.80E − 01
20	Flatness-shape	Radiomics		0.07	2.73E − 01	0.41	1.28E − 03	0.06	9.80E − 01
21	skewness – first order	Radiomics	SARS-CoV-2 < Control	0.47	2.29E − 03	0.06	3.37E − 01	0.01	9.80E − 01
22	Voxel volume-shape	Radiomics	SARS-CoV-2 < Control	0.48	2.28E − 03	0.23	3.80E − 02	0.01	9.80E − 01
23	Small area high gray level emphasis-glszm	Radiomics		0.16	8.42E − 02	0.06	3.37E − 01	0.01	9.80E − 01
24	Sphericity-shape	Radiomics	SARS-CoV-2 < Control	0.26	2.88E − 02	0.15	8.97E − 02	0.06	9.80E − 01
25	Dependence entropy-gldm	Radiomics	SARS-CoV-2 > Control	0.39	5.67E − 03	0.12	1.64E − 01	0.04	9.80E − 01
26	Sum average-glcm	Radiomics	SARS-CoV-2 > Control	0.45	2.83E − 03	0.22	3.80E − 02	0.00	9.80E − 01
27	Interquartile range – first order	Radiomics	SARS-CoV-2 > Control	0.49	2.28E − 03	0.11	1.74E − 01	0.02	9.80E − 01
28	Gray level non-uniformity-gldm	Radiomics	SARS-CoV-2 < Control	0.58	7.29E − 04	0.21	3.80E − 02	0.00	9.80E − 01
29	Mean-first order	Radiomics	SARS-CoV-2 > Control	0.44	2.83E − 03	0.22	3.80E − 02	0.00	9.80E − 01
30	Small dependence high gray level Emphasis-gldm	Radiomics	SARS-CoV-2 > Control	0.27	2.72E − 02	0.10	1.91E − 01	0.01	9.80E − 01
31	Gray Level Non-Uniformity-glszm	Radiomics		0.19	6.23E − 02	0.07	3.37E − 01	0.01	9.80E − 01
32	Root Mean Squared-first order	Radiomics	SARS-CoV-2 > Control	0.39	5.35E − 03	0.13	1.47E − 01	0.03	9.80E − 01
33	Size Zone Non-Uniformity-glszm	Radiomics	SARS-CoV-2 < Control	0.21	4.50E − 02	0.06	3.37E − 01	0.03	9.80E − 01
34	Surface Area-shape	Radiomics	SARS-CoV-2 < Control	0.30	1.91E − 02	0.10	1.91E − 01	0.05	9.80E − 01
35	Maximum 2D Diameter Column-shape	Radiomics	SARS-CoV-2 < Control	0.27	2.72E − 02	0.26	3.80E − 02	0.02	9.80E − 01

*mRMR* minimum redundancy maximum relevance; *ANOVA* analysis of variance; *ngtdm* neighboring gray-tone difference matrix; *glszm* gray-level size zone matrix; *glrlm* gray-level run length matrix; *glcm* gray-level co-occurrence matrix; *gldm* gray-level dependence matrix.

Mixed-effects ANOVAs were performed on all selected features to determine the effect of class and time.

Features are ordered by mRMR-permute rank.

The partial eta squared (η_p_^2^) and *p* value for the effects of class, time, and the interaction between class and time are given.

The direction of the mean difference between classes averaged across time is given for features with significant class effects (*p* value corrected for the false-discovery rate [*p*_fdr_] < 0.05).

All *p*_fdr_ values were attained using the Benjamini–Hochberg correction on the 35 comparisons.

**Figure 3:**
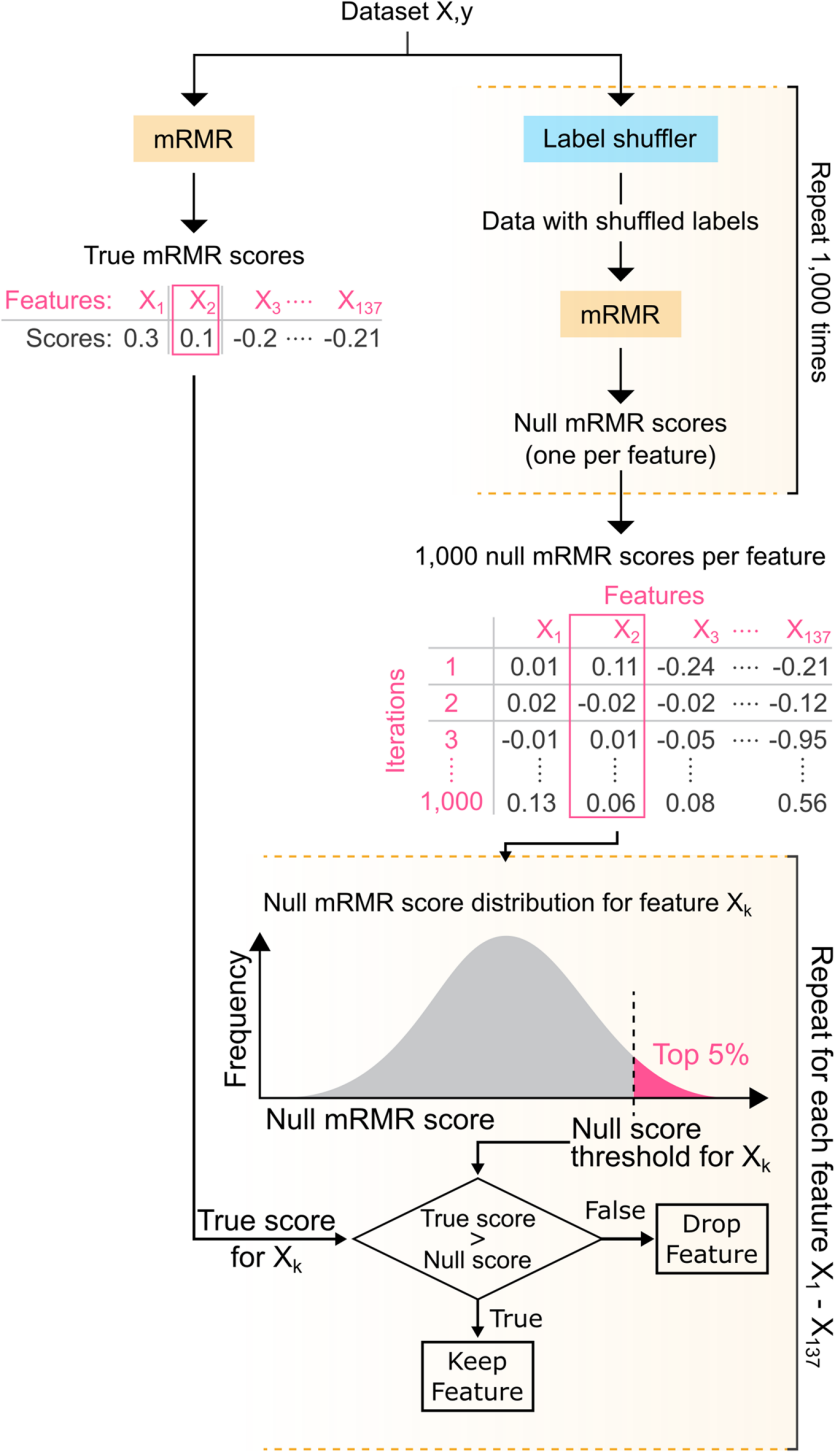
mRMR-permute. mRMR-permute combines mRMR and permutation testing to apply a statistical threshold for mRMR scores. mRMR score thresholds are based on null distributions created by permutating mRMR 1000 times with randomly shuffled labels. Once a null distribution is generated, a *p* value threshold of 0.05 can be applied to exclude features (X_k_) that do not exceed the null score threshold. mRMR, minimum redundancy maximum relevance.

The mRMR-permute feature-selection technique (Fig. 3) selected 35 features. These features consisted of 3 clinical pathological and 32 radiomic features. The 3 clinical pathological features ranked in the top 35% of mRMR-permute features. Although mRMR does not calculate a feature set size threshold, the performance of mRMR with a feature set size threshold of 35 was included in this study for comparison to mRMR-permute.

#### Feature set and model optimization

**Figure 4:**
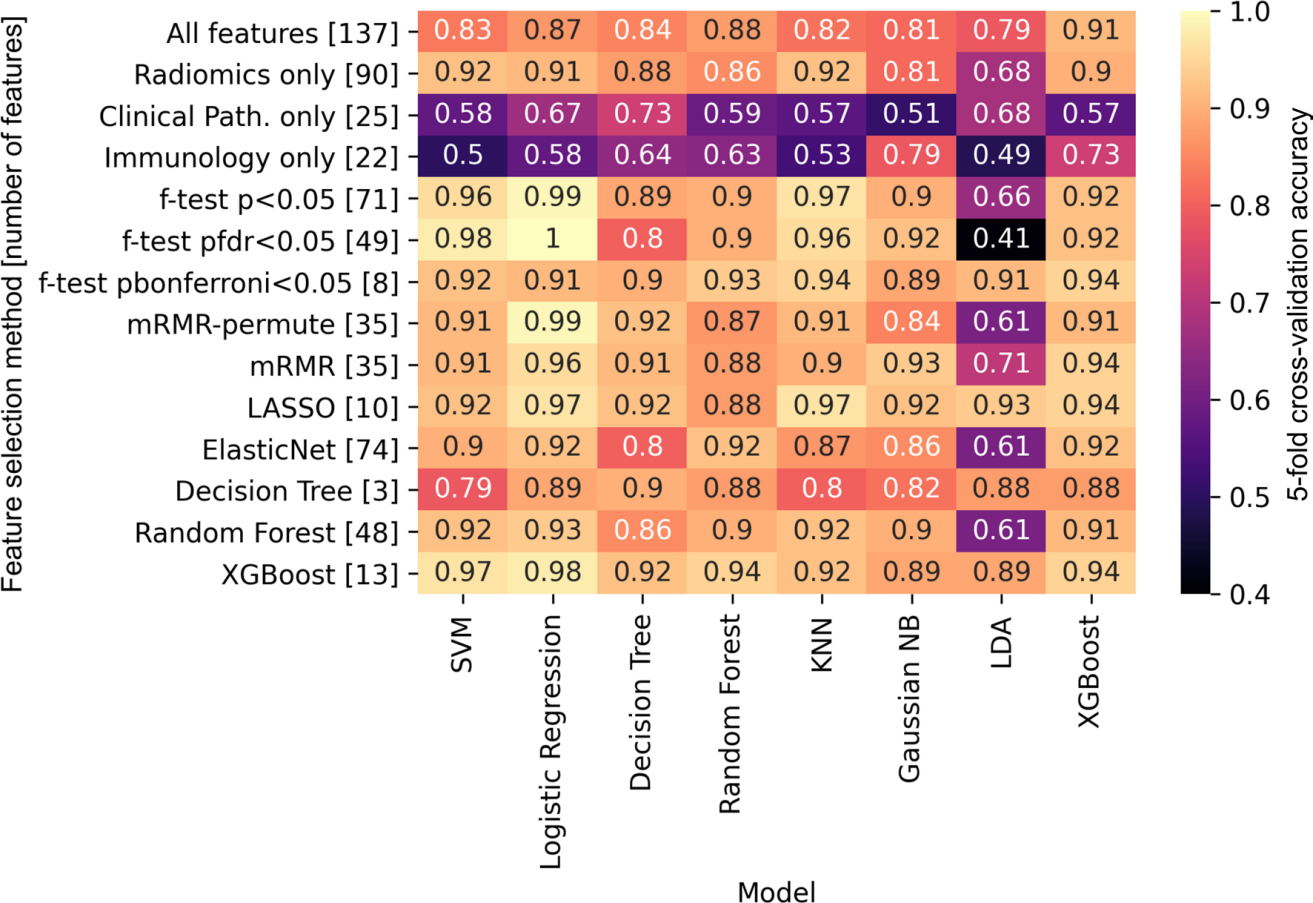
Feature set and model accuracy matrix. A total of 14 feature sets (10 feature-selection techniques, 3 single-domain feature sets, and 1 feature set containing all features) were used to train 8 models. The resulting fivefold stratified cross-validation accuracy was calculated and is shown within each cell of the matrix. The size of each feature set is given in brackets next to the feature selection method technique name. *p*_fdr_—*p* value corrected for the false-discovery rate; *p*_bonferroni_—*p* value after Bonferroni correction for multiple comparisons; *mRMR*—minimum redundancy maximum relevance; *LASSO*—least absolute shrinkage and selection operator; *SVM*—support vector machine; *KNN*—k-nearest neighbors; *NB*—naïve Bayes; *LDA*—linear discriminant analysis.

A total of 14 feature sets (10 feature-selection techniques, 3 single-domain feature sets, and 1 feature set that included all 137 features) were paired with 8 machine-learning models to form 112 trained models. Figure 4 shows a summary of the fivefold cross-validation accuracy of each feature set and model pair. Across all models, class-effect *p* value thresholds of *p* < 0.05, *p*_fdr_ < 0.05, and *p*_bonferroni_ < 0.05 had maximum accuracies of 0.99, 1.00, and 0.94, respectively. Scores from regression-based feature-selection techniques (LASSO and ElasticNet) had maximum accuracies across all models of 0.97 and 0.92, respectively. Scores from tree-based feature-selection techniques (decision tree, random forest, and XGBoost) had maximum accuracies across all models of 0.90, 0.93, and 0.98, respectively.

One feature set that included all features and 3 single-domain feature sets (radiomics-only [k = 90], clinical pathological-only [k = 25], and immunological-only [k = 22]), were included in this study to provide points of reference and for comparison to data reported in the literature. The feature set including all features had a maximum accuracy across all models of 0.91. The radiomic-only, clinical pathological-only, and immunological-only feature sets had maximum accuracies (across all models) of 0.92, 0.73, and 0.79, respectively.

When used to train a logistic regression model, 3 feature sets (a class-effect threshold of *p*_fdr_ < 0.05, a class-effect threshold of *p* < 0.05, and mRMR-permute) produced fivefold cross-validation accuracies equal to or greater than 0.99. Of these feature sets, the mRMR-permute feature set had the fewest features and was thus chosen for further analyses.

#### Model performance and analysis of selected features

mRMR-permute selected 35 out of 137 features and, when used to train a logistic regression model, produced fivefold cross-validation accuracies of 0.99 ± 0.04, precision-recall function (f1) scores of 0.97 ± 0.11, and receiver operating characteristic (ROC) curve area under the curves (AUCs) of 1.00 ± 0.00 (mean ± SD). Although a high model performance may suggest that the features in the feature set are predictive of SARS-CoV-2 infection, rigorous statistical methods are needed for validation. Therefore, the features selected by mRMR-permute were further analyzed to characterize the first-order effects of class, time, and the interaction between the effects of class and time (Table 2). All but 4 features had a significant class effect (mean partial eta squared [η_p_^2^] = 0.42; *p*_fdr_ < 0.05), 12 features had a significant time effect (mean η_p_^2^ = 0.27; *p*_fdr_ < 0.05), and no features had a significant interaction effect between class and time (*p*_fdr_ > 0.05). Longitudinal plots on mRMR-permute features are shown in Supplemental Fig. 1.

## Discussion

In this study, quantitative data, derived from chest CT images and blood sample analyses, were used to train a machine-learning algorithm to classify SARS-CoV-2 infection with clinically inapparent disease NHP model. Using a novel and statistically-thresholded feature-selection technique, mRMR-permute, a high classification accuracy (0.99 ± 0.04) was achieved. The mRMR-permute feature set consisted of 32 radiomic and 3 clinical pathological features, and we showed that 31 out of 35 features (89%) had a strong and significant infection class effect (mean η_p_^2^ = 0.42; *p*_fdr_ < 0.05).

Here, we proposed the application of permutation testing to calculate a statistical threshold for mRMR scores. This resulted in a small increase (0.03) in accuracy compared to mRMR with a matching feature set size used to train a matching model (logistic regression). However, the true strength of the technique is in the identification of a feature set size threshold using statistical methods not susceptible to user bias. Published variants of mRMR feature selection have used mRMR as a pre-processing step before applying other feature-selection methods, such as particle swarm optimization^30^, imperialist competition algorithm^31^, artificial bee colony^32^, incremental feature selection^33^, and genetic algorithm^34^. However, many of these methods still require users to choose a feature set size threshold or are used as wrappers (i.e., adjusted iteratively based on model performance), making them model-dependent.

To our knowledge, no published studies have examined the application of machine-learning techniques that incorporate radiomic analyses to classify the response to SARS-CoV-2 infection in an NHP model. Classification of SARS-CoV-2 infection in humans has largely focused on either radiomic features^35–39^ or blood biomarkers^40–42^, but rarely both. Homayounieh et al. trained logistic regression models on radiomic and clinical (i.e., demographics, symptoms, and laboratory data) features without feature selection (likely leading to high model complexity and low model generalizability) from a human dataset^43^. We expanded on this work by using a multimodal dataset and surveying 10 feature-selection methods and 8 models to predict SARS-CoV-2 infection in a clinically silent and highly controlled crab-eating macaque model. Homayounieh and colleagues reported that radiomic features outperformed clinical features in the prediction of the extent of pulmonary involvement, type of pulmonary opacities, and patient outcomes^43^. Similarly, we found that a logistic regression model trained on radiomic features outperformed one trained on clinical pathological features and immunological features in predicting infection.

When used to train machine-learning models, features selected by mRMR-permute produced classification accuracies as high as 0.99 ± 0.04. This suggests that the features chosen by mRMR-permute encompassed the information necessary for the classification of viral infection. We confirmed linear relationships between individual features and infection class in 31 out of 35 features using false-discovery rate-corrected mixed-effects ANOVAs. It is important to note that mRMR and mRMR-permute aim to maximize relevance and minimize redundancy; i.e., features that were excluded may have had a high relevance with the infection class but also a high redundancy with the other features. Additionally, mRMR-permute and mRMR rank relevance based on mutual information between features and class (virus-infected versus control) without modeling the time effect. This ranking is useful for building a model that can generalize across 2–6 days post-exposure; however, features that have strong but time-dependent class effects (e.g., interferon-alpha [IFNA1]—class effect: η_p_^2^ = 0.42, *p*_fdr_ = 1.2 × 10^−2^; time effect: η_p_^2^ = 0.55, *p*_fdr_ = 4.7 × 10^−5^; interaction effect: η_p_^2^ = 0.45, *p*_fdr_ = 1.3 × 10^−3^) might be “lost” along the way. In summary, the mRMR-permute feature set was derived in a data-driven manner and produced a minimal but high-performing classification model. However, the inclusion/exclusion of certain features was conceptually surprising; this may be explained by the multiple factors stated above that differentiate mRMR-permute from an f-test for the effect of class.

The following clinical pathological features were chosen by mRMR-permute feature selection: blood urea nitrogen concentration, absolute basophil counts, and absolute reticulocyte counts. These 3 features had large and significant class effects (SARS-CoV-2 < control; η_p_^2^ ≥ 0.24; *p*_fdr_ < 0.05). The blood urea nitrogen concentration is a surrogate measure of the renal glomerular filtration rate and has been shown to predict normal versus abnormal CT in COVID-19 patients^44^. However, for an NHP model without evidence of hepatocyte injury (class effect on aspartate aminotransferase and alanine aminotransferase activities: *p*_fdr_ > 0.05), relatively reduced dietary protein intake and/or improved hydration compared to the control group are the most plausible physiologic explanations for reduced blood urea nitrogen. Basophils are a minor granulocyte subpopulation within the leukocyte population and have overlapping functions with mast cells, including roles in hypersensitivity and anti-parasitical reactions. Because basophils are so few in normal animals (0–60 per µL, less than 1% of the differential), even minor changes may appear statistically (but not biologically) significant. Reduced basophil counts have been previously associated with COVID-19 in humans^45^, and lower basophil counts were observed in virus-infected compared to control macaques. This is consistent with increased migration of these cells into tissues; nevertheless, a complete absence of circulating basophils is within normal range. Reticulocytes are a specific stage of immature anucleate red blood cells with residual RNA and mitochondria, and absolute numbers in the peripheral blood are therefore reflective of erythropoietic reactivity^46^. The relatively decreased reticulocyte count in virus-infected compared to control macaques is consistent with a mild bone marrow response to an acute viral infection. Macaques in both groups underwent regular blood draws of equivalent volumes, however the effect of time and the interaction between the effects of class and time on reticulocyte count were not significant (*p*_fdr_ > 0.05). Although this constellation of clinical pathological features may in fact prove informative in future studies, the magnitude and directions of changes observed in these studies are not considered biologically significant in isolation.

Out of 32 radiomic features selected by mRMR-permute, 9 were shape features, 7 were first-order features, 6 were gray-level size zone matrix (GLSZM) features, 4 were gray-level co-occurrence matrix (glcm), 3 were gray-level dependence matrix (GLDM), 2 were neighboring gray-tone difference matrix (NGTDM) features, and 1 was a gray-level run length matrix feature (GLRLM). Shape features were calculated based on the shape of the region of interest, which, in this case, included whole lungs. We showed that shape features’ voxel volume, maximum three-dimensional (3D) diameter, mesh volume, surface area, and maximum 2D diameter column were significantly affected by viral infection (*p*_fdr_ < 0.05) and had lower values in SARS-CoV-2-infected compared to control macaques. These findings broadly indicate that, when lung pressure was kept constant during intubation while CT scanning is performed, SARS-CoV-2-infected animals had reduced lung volumes. This observation aligns with findings in humans of reduced total lung capacity compared to predicted values in COVID-19 patients and reduced percent predicted total lung capacity in severe cases compared to mild cases^47–49^. First-order features were calculated directly from the X-ray attenuation values measured in Hounsfield units (HUs). First-order features of increased mean, increased median, increased root mean squared, increased interquartile range, reduced skewness, reduced uniformity, and increased entropy (*p*_fdr_ < 0.05) were found in SARS-CoV-2-infected compared to control macaques. These measures broadly indicate hyperintensities and reduced homogeneity in CT scans of virus-infected macaques and align with previously reported high incidences of ground-glass opacities (77–98%) and consolidation (59–64%) in the lungs of COVID-19 patients^50–52^. Some features derived from filtered images (glszm, glcm, gldm, ngtdm, and glrlm) also align with clinical observations of COVID-19 patients. For example, GLSZM features are based on the size of zones (i.e., connected voxels that share the same intensity). We found reduced zone variance, size of zone nonuniformity, and large area emphasis (*p*_fdr_ < 0.05) in virus-infected macaques, broadly indicating that CT images of SARS-CoV-2-infected macaque lungs contained small equally sized zones. This interpretation is consistent with interlobar septal thickening observed in 59–75% of COVID-19 patients^50–52^. A full description of features from all 5 matrices can be found at https://pyradiomics.readthedocs.io/en/latest/features.html.

There are a few limitations of this study. Despite SARS-CoV-2 infection after exposure to high concentrations of the virus, clinical disease was, for the most part, inapparent. Therefore, our results may be specific to mild and subclinical disease due to SARS-CoV-2 infection and may not extrapolate to moderate or severe disease. If severe disease can be modeled in NHPs, the data presented here could be leveraged to improve model generalizability across a range of disease severities. Although we reported a high performance of the model, it is important to acknowledge that (as discussed above) this performance does not demonstrate that the input features are individually predictive of infection class. ANOVAs and *t*-tests were used in this study to statistically evaluate first-order relationships between each feature and the class variable. Finally, despite a relatively high sample size compared to most NHP studies (80 samples from 20 crab-eating macaques), this study had a relatively low sample size compared to most SARS-CoV-2 work in humans—highlighting the need for replication studies to confirm our findings. Future work should include further replicates, prioritizing macaques that better model severe disease in humans. This may be accomplished using models of medical conditions known to increase the risk of severe COVID-19 in humans such as advanced age, obesity, and chronic lung disease^53^.

In conclusion, we demonstrated that a logistic regression model trained on the mRMR-permute feature set achieved an accuracy of 0.99 ± 0.04 with fewer features than models of equal or higher accuracy. SARS-CoV-2 infection was predicted with a high degree of accuracy in an otherwise clinically “silent” model of disease. We then statistically characterized the mRMR-permute features relative to SARS-CoV-2 infection and showed that 31 out of 35 features had strong and significant virus infection effects after modeling the time effects. Classification of SARS-CoV-2 infection in a highly controlled NHP model represents a key first building block towards the classification of within-disease severity and, ultimately, prediction of the physiological response to infection.

## Materials and methods

### Dataset

This study utilizes a dataset first acquired and published in 2020 via a pre-print^24^; a full manuscript containing the primary findings from these experiments is in preparation. Experimental procedures for this study were approved by the National Institute of Allergy and Infectious Diseases (NIAID) Division of Clinical Research (DCR) Animal Care and Use Committee (ACUC) and were in compliance with the Animal Welfare Act regulations, Public Health Service policy, and the *Guide for the Care and Use of Laboratory Animals 8th Ed.* recommendations. All work was performed in accordance with the recommendations of the Weatherall Report. This study is reported in accordance with Animal Research: Reporting of In Vivo Experiments (ARRIVE) guidelines^54^. Animal care and experiments were performed at a maximum (biosafety level 4 [BSL-4]) containment laboratory at the NIH NIAID DCR Integrated Research Facility at Fort Detrick (IRF-Frederick), a facility accredited by the Association for Assessment and Accreditation of Laboratory Animal Care International (AAALAC). A total of 20 crab-eating macaques (*Macaca fascicularis* Raffles, 1821), with an age range of 4.0–6.8 years, were exposed to either severe acute respiratory syndrome coronavirus 2 (SARS-CoV-2 group; n = 12) or a mock inoculum (control group; n = 8). Virus and mock exposures were administered either intra-bronchially or through inhalation of aerosolized particles. Infection and absence of infection were confirmed by testing for neutralizing anti-SARS-CoV-2 antibodies in sera, and viral nucleic acid in swab material collected before and after exposures. Sex, country of origin, and the ratio of intra-bronchial to aerosol exposure were matched across SARS-CoV-2-infected and control groups. The age, sex, country of origin, weight, change in weight, exposure route, and peak clinical scores are shown in Table 1. CT scans and blood samples were collected at pre-exposure and 2, 4, and 6 days post-exposure, resulting in a total of 80 samples per measure. The heterogeneity of the exposure routes creates a more difficult classification task but improves model generalizability. A diagram of the time points of each data collection relative to the date of exposure is provided in Fig. 1A. The virus and mock intrabronchial exposure procedures were previously described in detail^24^. Complete genome sequences of the SARS-CoV-2 isolates were published by NCBI (GenBank MW161259 and MT952134).

### Non-imaging data collection

Although CT imaging can visualize lesions that result from SARS-CoV-2 infection of lung tissue, non-imaging measures have the potential to detect changes in the downstream and upstream processes of lung damage. For example, cageside assessments capture disruptions in lung function that result from lung tissue damage, whereas measures of cytokine activity quantify the immune system reaction to infection, resulting in lesion formation. Previously described cageside assessment scoring criteria^24^ were modified from previous work^55^ to include clinical signs relevant to COVID-19 and respiratory rates of crab-eating macaques^56^.

Blood sample processing procedures used to generate clinical pathological and immunological measures were previously described in detail^24^, however, key points are provided here. Complete blood cell counts, including leukocyte differentials and reticulocyte counts, were determined from blood samples using the Procyte DX (IDEXX Laboratories, Westbrook, ME, USA). The Catalyst One analyzer (IDEXX Laboratories) was used for biochemical analyses of serum samples. Seven Catalyst tests were used to generate data for the following parameters: blood urea nitrogen concentration, alanine aminotransferase activity, albumin concentration, aspartate aminotransferase activity, creatinine concentration, lactate dehydrogenase activity, and total protein concentration. Globulin concentration and albumin-globulin ratio were calculated from these results.

Immunological measures were generated from 25-µL plasma samples using the MILLIPLEX MAP Non-Human Primate Cytokine Magnetic Bead Panel – Premixed 23 Plex – Immunology Multiplex Assay (Millipore, Burlington, MA, USA; #PCYTMG-40K-PX23) according to the manufacturer’s instructions and were read on a Flexmap 3D reader (Luminex, Chicago, IL, USA) within 24 h of completion following assay instructions. Data was exported to Bio-Results Generator version 3.0 and Bio-Plex Manager software version 6.2 (BioRad).

### CT image collection and radiomic feature extraction

CT images were collected on a Philips Gemini 16-slice CT scanner (Philips Healthcare, Cleveland, OH, USA) in helical scan mode. The scanner parameters were set at 140 kVp, 300 mA per slice, 1-mm thickness, 0.5-mm increment, 0.688-mm pitch, collimation 16 × 0.75, and 0.75 s rotation. CT image reconstruction used a 512 × 512 matrix for a 250-mm transverse field of view (pixel size = 0.488 mm). Each macaque was subject to a 15–20 s breath-hold during acquisition. The pressure of the breath-hold was maintained at 150 mm H_2_O.

Whole-lung masks of the CT images were generated using a deep-learning neural network called a feature pyramids network^57^. Full details on the model architecture and training parameters have been described previously^58,59^. The feature pyramids network excels at recognizing objects across a large range of scales by using 2 pyramidal pathways to build high-level semantic feature maps at 4 scales (1/4, 1/8, 1/6, and 1/32). The loss function used was the focal loss and the key training parameters were as follows: 128 × 128 × 16 patch size, Adam optimizer, 5 × 10^−5^ learning rate, 5 × 10^−5^ decay rate, 30 epochs, 1 × 10^−5^ minimum delta for early stopping, and patience on validation accuracy of 4. Representative images of the segmentation performance on infected and uninfected lungs are shown in Fig. 1B. Morphological features were extracted from whole-lung masked CT images using PyRadiomics 2.20^60^ within 3D Slicer^61^ (https://www.slicer.org/). For each image, 111 features were extracted: 17 3D shape features, 19 first-order intensity features, and 75 second-order intensity features. The second-order features were derived from 5 different matrices: (1) 24 features from the gray-level co-occurrence matrix (GLCM); (2) 14 features from the gray-level dependence matrix (GLDM); (3) 16 features from the gray-level run length matrix (GLRLM); (4) 16 features from the gray-level size zone matrix (GLSZM); (5) 5 features from the neighboring gray tone difference matrix (NGTDM).

### Dimensionality reduction

#### Exploratory feature correlation analysis

Pearson’s correlation coefficient was calculated among all features to assess macro trends in the data and determine if dimensionality reduction was necessary. These results are presented graphically in a correlation matrix in Fig. 1C. The average absolute magnitude of Pearson’s correlation coefficient was calculated across features intra-domain (e.g., radiomic features correlated with radiomic features) and inter-domain (e.g., radiomic features correlated with immunological features). Additionally, the percentage of correlations with coefficients greater than 0.7 (a common threshold for a strong correlation^28^) was reported to highlight groups of correlations with high rates of redundant features. When aggregating correlation coefficients, duplicate correlations (e.g., albumin versus interleukin 2 [IL2] and IL2 versus albumin) and correlations between identical features (e.g., albumin versus albumin) were excluded.

#### Domain-specific feature screening

A total of 170 radiomic, clinical pathological, and immunological features were derived from CT images and blood analyses. A full list of features stratified by domain as well as inclusion/exclusion from model training is provided in Supplemental Material 1. This feature set was screened using domain-specific criteria set by subject-matter experts (co-authors M.A.C., S.M.A., and T.K.C.). By applying domain-specific criteria, subject-matter expertise can be leveraged to ensure the resultant model has a scientific basis, leading to better generalizability. The resulting feature dropout is summarized in Fig. 2.

Radiomic features were screened for stability and sensitivity using a procedure outlined in previous work^29^. For each radiomic feature, the normal variation (NV) was calculated as the inter-subject maximum (across control macaques) of the intra-subject maximum variation relative to their baseline scan. The dynamic range (DR) of each feature due to SARS-CoV-2 infection was calculated as the inter-subject average (across SARS-CoV-2-infected macaques) of the intra-subject maximum variation relative to their baseline scan. For a given feature *i*, a ratio R_*i*_ = (DR_*i*_–NV_*i*_)*100/DR_*i*_ was computed to characterize the sensitivity of each feature when compared to the normal variation. Ratios near 0 indicate that the dynamic range is comparable to the normal variation, whereas ratios close to 100% indicate that those features are very sensitive to the disease compared to the normal variation. Features with ratios below 0% were screened from further analysis.

Clinical pathological features were removed if they were directly derived from other features. For example, the absolute monocyte count was preferred to the percent monocytes because the percent monocytes is relative to the total cell count and, thus, has little interpretable value on its own.

### Data-driven feature selection

Following domain-specific feature screening, the feature set was further reduced in a data-driven manner. A total of 10 feature-selection techniques were employed and included both model-independent and model-dependent techniques. A mixed-effects ANOVA analyzing the effects of class (SARS-CoV-2-infected versus control) and time (2, 4, and 6 days post-exposure change from baseline) was performed for each feature. The f-test results for the effect of group were used as feature-selection criteria. A total of 3 *p* value thresholds were examined in this study: *p* < 0.05, *p* < 0.05 after false discovery rate correction (Benjamini–Hochberg), and *p* < 0.05 after Bonferroni correction for multiple comparisons (i.e., *p* < 3.6 × 10^−4^).

Six model-dependent feature-selection techniques were examined. Logistic-regression feature-selection techniques were implemented with 2 different regularization methods: least absolute shrinkage and selection operator (LASSO) and Elastic net (L1 ratio = 0.5). Three tree-based feature-importance techniques were also implemented for feature selection (using an importance threshold of 0): Decision Tree, Random Forest, and XGBoost. These feature-selection techniques were implemented using the scikit-learn (version 0.23.2) library for Python^62^ with default options.

### mRMR-permute

In this study, we proposed and applied a novel feature-selection technique that combines the minimum redundancy maximum relevance technique (mRMR)^27^ with permutation testing. This technique leverages the strengths of mRMR to rank features but removes the need to choose a feature set size threshold. In the selection of a feature set size threshold, scientists may introduce bias into the predictive model, leading to poor generalizability. The procedure for mRMR permute is outlined in Fig. 3 and is described here. First, mRMR was run on the dataset to generate the true mRMR scores for each feature. Permutation testing was performed by generating a set of null mRMR scores (one per feature) by randomly shuffling the class labels and running mRMR on the shuffled dataset. This procedure was then repeated 1,000 times to create 1,000 null mRMR scores per feature. Each feature’s null mRMR scores were used to build a null mRMR score distribution for that feature. A *p* value threshold of 0.05 was then applied to the null distribution to calculate the mRMR score threshold for each feature. Although some have proposed stricter statistical thresholds for use in clinical research^63^, a *p* value of 0.05 may be acceptable for this application, given that the cost of false positives (features incorrectly included in the feature set) is not hidden but instead quantified by measures of model performance. Our implementation applied the *p* value threshold in a discrete manner by taking the lowest of the top 5% of scores (the fiftieth highest score out of 1000 null scores) to be the null mRMR score threshold. If the true mRMR score for a feature did not exceed the null mRMR score threshold for that feature, the feature was eliminated. mRMR-permute features were therefore ranked in the same order as mRMR rankings but with non-significant features removed. In this study, mRMR-permute selected 35 out of 137 features. The performance of mRMR with a feature set size threshold of 35 was included in this study for comparison.

### Machine-learning model development

Eight popular machine-learning models were implemented in this study using the scikit-learn (version 0.23.2) Python toolbox^62^. These models included support vector machine, k-nearest neighbors, Gaussian naïve Bayes, logistic regression, decision tree, random forest, XGBoost^64^, and linear discriminant analysis. A total of 14 feature sets (10 feature-selection methods, 3 single-domain feature sets, and a set including all features) were paired with each of the 8 machine-learning models. The fivefold stratified cross-validation accuracy was calculated for each of the 112 model combinations. Each fold was stratified to ensure that macaques were unique to either the testing or training group.

The final model and feature-selection technique was chosen to be the smallest feature set with a fivefold stratified cross-validation accuracy above 0.99. The performance of the final model was assessed in detail using the fivefold stratified cross-validation f1 score and the area under the curve (AUC) of the receiver operator characteristic curve.

### Statistical analysis

Welch’s *t*-test and Pearson’s chi-squared test were used to test for between-group differences in demographic features (age, sex, country of origin, inoculation mode, weight, average change in weight, and maximum clinical score). Feature selection and model training were performed on data relative to each animal’s baseline measurement for that feature. Mixed-effects ANOVAs were performed to assess the effect of group (between-unit) and time (within-unit) on the selected features. Subsequently, *p* values for the group, time, and the interaction between group and time effects were corrected for the false discovery rate^65^, and the partial eta squared is reported to quantify effect size.

### Supplementary Information

Supplementary Information.

## Data Availability

The datasets generated during and/or analyzed during the current study are available from the corresponding author upon reasonable request.

## Code availability

Our code is publicly available at: https://github.com/wtchu8/IRF_ML_SARSCoV2_pub.
