# Supplementary material for: Novel machine-learning analysis of SARS-CoV-2 infection in a subclinical nonhuman primate model using radiomics and blood biomarkers
Source: Sci Rep. 2023 Nov 10;13:19607. doi: 10.1038/s41598-023-46694-9 (PMC10638262; doi:10.1038/s41598-023-46694-9)
Supplement: Supplementary file 1 — Supplementary Information. [file 41598_2023_46694_MOESM1_ESM.pdf]

Supplemental Information

Novel machine-learning analysis of SARS-CoV-2 in a subclinical nonhuman primate model using radiomics and blood biomarkers

Winston T. Chu, Marcelo A. Castro, Syed Reza, Timothy K. Cooper, Sean Bartlinski, Dara Bradley, Scott M. Anthony, Gabriella Worwa, Courtney L. Finch, Jens H. Kuhn, Ian Crozier, Jeffrey Solomon*
*Corresponding author

Supplemental Table 1: Data-driven feature selection.

Feature Selection	Radiomic	Clinical Pathology	Immunology	Total	Top-performing model(s)
f-test class effect $p < 0.05$	63	4	4	71	Logistic regression
f-test class effect $p_{\text{fdr}} < 0.05$	44	2	3	49	Logistic regression
f-test class effect $p_{\text{bonferroni}} < 0.05$	8	0	0	8	kNN
Logistic regression - LASSO	6	4	0	10	kNN, Logistic regression
Logistic regression - ElasticNet	52	16	6	74	Logistic regression, Random forest, XGBoost
Decision tree	1	2	0	3	Decision tree
Random forest	44	4	0	48	Logistic regression
XGBoost	8	3	2	13	Logistic regression
mRMR-permute	32	3	0	35	Logistic regression
mRMR (k=35)	23	6	6	35	Logistic regression

Distribution of features across three domains: radiomics, clinical pathological, and immunological. p_{fdr}, p-value after correction for the false discovery rate; $p_{\text{bonferroni}}$, p-value after applying the Bonferroni correction. LASSO, least absolute shrinkage and selection operator; mRMR, minimum redundancy maximum relevance; kNN, k-nearest neighbors.

Supplemental Table 2: Class–time ANOVA for all features.

Feature	Modality	Class effect			Time effect		Class–time interaction effect	
		Direction	η_p^2	p_{fdr}	η_p^2	p_{fdr}	η_p^2	p_{fdr}
Gray Level Non-Uniformity-gldm	Radiomics	SARS-CoV-2<Control	5.83E-01	2.85E-03	2.14E-01	6.16E-02	4.59E-03	9.58E-01
Uniformity-first order	Radiomics	SARS-CoV-2<Control	6.37E-01	2.85E-03	2.35E-01	5.54E-02	6.80E-03	9.57E-01
Zone Variance-glszm	Radiomics	SARS-CoV-2<Control	5.76E-01	2.85E-03	1.01E-01	2.79E-01	3.06E-02	9.07E-01
Large Area Emphasis-glszm	Radiomics	SARS-CoV-2<Control	5.76E-01	2.85E-03	1.01E-01	2.79E-01	3.06E-02	9.07E-01
Busyness-ngtdm	Radiomics	SARS-CoV-2<Control	6.01E-01	2.85E-03	9.64E-02	2.83E-01	2.01E-02	9.07E-01
IDMN-glcm	Radiomics	SARS-CoV-2>Control	5.63E-01	3.15E-03	5.07E-02	5.15E-01	7.22E-03	9.57E-01
Entropy-first order	Radiomics	SARS-CoV-2>Control	5.34E-01	4.29E-03	1.78E-01	1.18E-01	2.09E-02	9.07E-01
Gray Level Non-Uniformity Normalized-glrlm	Radiomics	SARS-CoV-2<Control	5.40E-01	4.29E-03	1.78E-01	1.18E-01	1.61E-02	9.07E-01
Joint Entropy-glcm	Radiomics	SARS-CoV-2>Control	5.02E-01	6.48E-03	1.55E-01	1.55E-01	1.08E-02	9.24E-01
Sum Entropy-glcm	Radiomics	SARS-CoV-2>Control	5.03E-01	6.48E-03	1.86E-01	1.12E-01	2.44E-02	9.07E-01
Voxel Volume-shape	Radiomics	SARS-CoV-2<Control	4.79E-01	7.02E-03	2.26E-01	5.86E-02	1.22E-02	9.24E-01
Mesh Volume-shape	Radiomics	SARS-CoV-2<Control	4.79E-01	7.02E-03	2.26E-01	5.86E-02	1.23E-02	9.24E-01
Interquartile Range-first order	Radiomics	SARS-CoV-2>Control	4.90E-01	7.02E-03	1.14E-01	2.48E-01	1.67E-02	9.07E-01
Large Area Low Gray Level Emphasis-glszm	Radiomics	SARS-CoV-2<Control	4.81E-01	7.02E-03	1.65E-01	1.40E-01	3.43E-02	9.07E-01
Skewness-first order	Radiomics	SARS-CoV-2<Control	4.74E-01	7.17E-03	6.17E-02	4.49E-01	1.11E-02	9.24E-01
Median-first order	Radiomics	SARS-CoV-2>Control	4.68E-01	7.57E-03	4.13E-01	1.43E-03	5.96E-02	9.07E-01
Joint Average-glcm	Radiomics	SARS-CoV-2>Control	4.45E-01	9.43E-03	2.20E-01	5.86E-02	1.13E-03	9.80E-01
Joint Energy-glcm	Radiomics	SARS-CoV-2<Control	4.45E-01	9.43E-03	2.50E-01	4.54E-02	2.74E-03	9.79E-01
Sum Average-glcm	Radiomics	SARS-CoV-2>Control	4.45E-01	9.43E-03	2.20E-01	5.86E-02	1.13E-03	9.80E-01
Mean-first order	Radiomics	SARS-CoV-2>Control	4.39E-01	9.48E-03	2.18E-01	5.90E-02	1.24E-03	9.80E-01
Blood Urea Nitrogen (mg/dL)	Clinical Pathology	SARS-CoV-2<Control	4.39E-01	9.48E-03	1.82E-01	1.14E-01	2.73E-02	9.07E-01
IL6R (pg/mL)	Immunology	SARS-CoV-2>Control	4.36E-01	9.50E-03	5.46E-01	4.67E-05	4.42E-01	1.28E-03
Idn-glcm	Radiomics	SARS-CoV-2>Control	4.24E-01	1.11E-02	2.95E-02	6.34E-01	4.89E-03	9.58E-01
IFNA1 (pg/mL)	Immunology	SARS-CoV-2>Control	4.18E-01	1.18E-02	5.47E-01	4.67E-05	4.46E-01	1.28E-03
IL15 (pg/mL)	Immunology	SARS-CoV-2>Control	4.16E-01	1.18E-02	2.35E-01	5.54E-02	3.24E-01	2.00E-02
Gray Level Non-Uniformity-glrlm	Radiomics	SARS-CoV-2<Control	4.11E-01	1.23E-02	1.45E-01	1.78E-01	8.30E-03	9.57E-01
Robust Mean Absolute Deviation-first order	Radiomics	SARS-CoV-2>Control	4.01E-01	1.39E-02	1.05E-01	2.78E-01	3.79E-02	9.07E-01
Contrast-ngtdm	Radiomics	SARS-CoV-2<Control	3.97E-01	1.43E-02	3.30E-02	6.03E-01	7.31E-03	9.57E-01
Root Mean Squared-first order	Radiomics	SARS-CoV-2>Control	3.94E-01	1.44E-02	1.26E-01	2.28E-01	2.66E-02	9.07E-01
Dependence Entropy-gldm	Radiomics	SARS-CoV-2>Control	3.87E-01	1.55E-02	1.18E-01	2.44E-01	4.03E-02	9.07E-01
Low Gray Level Emphasis-gldm	Radiomics	SARS-CoV-2<Control	3.84E-01	1.57E-02	2.32E-01	5.62E-02	4.01E-02	9.07E-01
Autocorrelation-glcm	Radiomics	SARS-CoV-2>Control	3.74E-01	1.72E-02	1.14E-01	2.48E-01	2.86E-02	9.07E-01
High Gray Level Emphasis-gldm	Radiomics	SARS-CoV-2>Control	3.75E-01	1.72E-02	1.17E-01	2.45E-01	2.67E-02	9.07E-01
Large Area High Gray Level Emphasis-glszm	Radiomics	SARS-CoV-2<Control	3.56E-01	2.20E-02	4.22E-02	5.44E-01	2.76E-02	9.07E-01
90 Percentile-first order	Radiomics	SARS-CoV-2>Control	3.50E-01	2.29E-02	9.90E-02	2.80E-01	3.90E-02	9.07E-01
Kurtosis-first order	Radiomics	SARS-CoV-2<Control	3.50E-01	2.29E-02	2.42E-02	6.88E-01	2.20E-02	9.07E-01
10 Percentile-first order	Radiomics	SARS-CoV-2>Control	3.39E-01	2.61E-02	3.58E-01	4.32E-03	1.05E-01	7.40E-01
Low Gray Level Run Emphasis-glrlm	Radiomics	SARS-CoV-2<Control	3.34E-01	2.62E-02	1.72E-01	1.31E-01	3.86E-02	9.07E-01
Coarseness-ngtdm	Radiomics	SARS-CoV-2>Control	3.34E-01	2.62E-02	1.12E-01	2.53E-01	2.81E-02	9.07E-01
Absolute Basophil ($10^3/\mu L$)	Clinical Pathology	SARS-CoV-2<Control	3.34E-01	2.62E-02	1.57E-01	1.53E-01	2.92E-01	3.38E-02
Least Axis Length-shape	Radiomics	SARS-CoV-2<Control	3.27E-01	2.79E-02	1.06E-02	8.44E-01	2.80E-02	9.07E-01
Range-first order	Radiomics	SARS-CoV-2>Control	3.25E-01	2.79E-02	4.42E-02	5.33E-01	2.48E-02	9.07E-01
Maximum-first order	Radiomics	SARS-CoV-2>Control	3.25E-01	2.79E-02	4.42E-02	5.33E-01	2.48E-02	9.07E-01
High Gray Level Run Emphasis-glrlm	Radiomics	SARS-CoV-2>Control	3.15E-01	3.13E-02	8.54E-02	3.31E-01	3.08E-02	9.07E-01
Short Run High Gray Level	Radiomics	SARS-CoV-2>Control	3.08E-01	3.39E-02	8.49E-02	3.31E-01	2.99E-02	9.07E-01

Emphasis-glrlm								
Surface Area-shape	Radiomics	SARS-CoV-2<Control	2.99E-01	3.73E-02	1.01E-01	2.79E-01	4.95E-02	9.07E-01
Long Run High Gray Level Emphasis-glrlm	Radiomics	SARS-CoV-2>Control	2.92E-01	4.05E-02	4.67E-02	5.31E-01	2.15E-02	9.07E-01
Large Dependence High Gray Level Emphasis-gldm	Radiomics	SARS-CoV-2>Control	2.81E-01	4.65E-02	2.09E-02	7.21E-01	4.44E-03	9.58E-01
Total Energy-first order	Radiomics	SARS-CoV-2>Control	2.74E-01	4.97E-02	7.83E-02	3.59E-01	6.17E-02	9.07E-01
Maximum 3D Diameter-shape	Radiomics		2.68E-01	5.21E-02	3.86E-01	2.64E-03	3.10E-02	9.07E-01
Maximum 2D Diameter Column-shape	Radiomics		2.68E-01	5.21E-02	2.57E-01	4.40E-02	1.60E-02	9.07E-01
Small Dependence High Gray Level Emphasis-gldm	Radiomics		2.65E-01	5.32E-02	1.04E-01	2.78E-01	1.31E-02	9.24E-01
Compactness2-shape	Radiomics		2.54E-01	5.60E-02	1.69E-01	1.32E-01	5.86E-02	9.07E-01
Sphericity-shape	Radiomics		2.55E-01	5.60E-02	1.55E-01	1.55E-01	5.99E-02	9.07E-01
Compactness1-shape	Radiomics		2.56E-01	5.60E-02	1.58E-01	1.53E-01	5.97E-02	9.07E-01
Run Entropy-glrlm	Radiomics		2.56E-01	5.60E-02	5.01E-02	5.15E-01	6.73E-02	9.07E-01
CCL2 (pg/mL)	Immunology		2.58E-01	5.60E-02	4.96E-01	1.99E-04	4.41E-01	1.28E-03
Spherical Disproportion-shape	Radiomics		2.47E-01	6.05E-02	1.38E-01	1.88E-01	5.94E-02	9.07E-01
Mean Absolute Deviation-first order	Radiomics		2.39E-01	6.35E-02	1.14E-01	2.48E-01	7.50E-02	9.07E-01
Short Run Low Gray Level Emphasis-glrlm	Radiomics		2.42E-01	6.35E-02	1.00E-01	2.79E-01	3.87E-02	9.07E-01
Small Area Emphasis-glszm	Radiomics		2.39E-01	6.35E-02	5.64E-02	4.88E-01	3.16E-02	9.07E-01
Absolute Reticulocyte (10^3/µL)	Clinical Pathology		2.41E-01	6.35E-02	3.39E-02	5.99E-01	9.61E-02	7.40E-01
Size Zone Non-Uniformity Normalized-glszm	Radiomics		2.34E-01	6.72E-02	5.47E-02	4.88E-01	3.07E-02	9.07E-01
Major Axis Length-shape	Radiomics		2.28E-01	7.15E-02	3.68E-01	3.59E-03	6.22E-03	9.57E-01
MCC-glcm	Radiomics		2.19E-01	7.93E-02	3.27E-02	6.03E-01	2.62E-02	9.07E-01
Minor Axis Length-shape	Radiomics		2.17E-01	7.96E-02	2.12E-02	7.21E-01	2.24E-01	1.20E-01
Size Zone Non-Uniformity-glszm	Radiomics		2.14E-01	8.15E-02	6.26E-02	4.46E-01	2.80E-02	9.07E-01
Energy-first order	Radiomics		2.10E-01	8.50E-02	9.31E-02	2.98E-01	1.01E-01	7.40E-01
High Gray Level Zone Emphasis-glszm	Radiomics		2.07E-01	8.53E-02	4.70E-02	5.31E-01	1.86E-02	9.07E-01
Platelet (10^3/µL)	Clinical Pathology		2.08E-01	8.53E-02	1.22E-01	2.33E-01	2.66E-01	5.26E-02
Correlation-glcm	Radiomics		2.01E-01	9.20E-02	1.31E-01	2.11E-01	1.05E-01	7.40E-01
Gray Level Non-Uniformity-glszm	Radiomics		1.87E-01	1.08E-01	6.78E-02	4.24E-01	1.10E-02	9.24E-01
IMC 2-glcm	Radiomics		1.81E-01	1.14E-01	9.76E-03	8.51E-01	4.82E-02	9.07E-01
CXCL8 (pg/mL)	Immunology		1.81E-01	1.14E-01	8.23E-02	3.44E-01	9.30E-02	7.62E-01
Strength-ngtdm	Radiomics		1.79E-01	1.15E-01	4.59E-02	5.32E-01	2.92E-02	9.07E-01
IMC 1-glcm	Radiomics		1.78E-01	1.15E-01	1.99E-02	7.28E-01	5.37E-02	9.07E-01
Cluster Tendency-glcm	Radiomics		1.75E-01	1.18E-01	1.41E-01	1.85E-01	1.11E-01	7.40E-01
Sum Squares-glcm	Radiomics		1.73E-01	1.19E-01	1.34E-01	2.03E-01	1.02E-01	7.40E-01
Maximum 2D Diameter Row-shape	Radiomics		1.69E-01	1.24E-01	3.74E-01	3.28E-03	4.30E-02	9.07E-01
Small Area High Gray Level Emphasis-glszm	Radiomics		1.61E-01	1.36E-01	6.34E-02	4.43E-01	5.85E-03	9.57E-01
Standard Deviation-first order	Radiomics		1.55E-01	1.46E-01	1.21E-01	2.33E-01	9.64E-02	7.40E-01
Gray Level Variance-gldm	Radiomics		1.49E-01	1.53E-01	1.22E-01	2.33E-01	9.85E-02	7.40E-01
Variance-first order	Radiomics		1.49E-01	1.53E-01	1.22E-01	2.33E-01	9.85E-02	7.40E-01
White Blood Cell (10^3/µL)	Clinical Pathology		1.44E-01	1.61E-01	5.44E-03	9.13E-01	2.04E-02	9.07E-01
Dependence Non-Uniformity-gldm	Radiomics		1.38E-01	1.72E-01	9.99E-02	2.79E-01	4.39E-02	9.07E-01
Albumin (g/dL)	Clinical Pathology		1.37E-01	1.72E-01	1.70E-01	1.32E-01	6.15E-02	9.07E-01
Zone Entropy-glszm	Radiomics		1.33E-01	1.80E-01	1.85E-01	1.12E-01	1.69E-01	3.47E-01
Run Length Non-Uniformity-	Radiomics		1.29E-01	1.87E-01	1.10E-01	2.61E-01	4.45E-02	9.07E-01

glrlm							
Cluster Prominence-glcm	Radiomics	1.25E-01	1.95E-01	1.53E-01	1.57E-01	1.22E-01	6.62E-01
Cluster Shade-glcm	Radiomics	1.18E-01	2.11E-01	1.59E-01	1.53E-01	1.29E-01	6.06E-01
Absolute Neutrophil (10^3/μL)	Clinical Pathology	1.14E-01	2.19E-01	2.83E-02	6.44E-01	1.11E-02	9.24E-01
Gray Level Variance-glrlm	Radiomics	1.12E-01	2.22E-01	1.17E-01	2.45E-01	1.03E-01	7.40E-01
Complexity-ngtdm	Radiomics	1.11E-01	2.22E-01	7.14E-02	4.06E-01	4.15E-02	9.07E-01
Gray Level Variance-glszm	Radiomics	1.03E-01	2.44E-01	6.63E-02	4.24E-01	6.94E-02	9.07E-01
Albumin-Globulin Ratio	Clinical Pathology	1.01E-01	2.49E-01	5.52E-02	4.88E-01	1.77E-02	9.07E-01
Platelet crit (%)	Clinical Pathology	9.85E-02	2.54E-01	1.43E-01	1.82E-01	3.24E-01	2.00E-02
Difference Variance-glcm	Radiomics	8.27E-02	3.09E-01	4.57E-02	5.32E-01	1.67E-02	9.07E-01
Mean Corpuscular Hemoglobin Concentration (g/L)	Clinical Pathology	7.53E-02	3.38E-01	8.60E-02	3.31E-01	2.66E-01	5.26E-02
Flatness-shape	Radiomics	6.64E-02	3.78E-01	4.11E-01	1.43E-03	6.43E-02	9.07E-01
IL18 (pg/mL)	Immunology	6.48E-02	3.82E-01	5.48E-02	4.88E-01	3.72E-02	9.07E-01
Absolute Lymphocyte (10^3/μL)	Clinical Pathology	6.38E-02	3.83E-01	2.56E-01	4.40E-02	3.53E-01	1.37E-02
IL6 (pg/mL)	Immunology	5.91E-02	4.05E-01	3.60E-02	5.90E-01	1.97E-02	9.07E-01
CCL3 (pg/mL)	Immunology	5.22E-02	4.43E-01	1.38E-01	1.88E-01	1.31E-01	6.06E-01
CCL4 (pg/mL)	Immunology	5.04E-02	4.50E-01	2.79E-01	3.14E-02	1.38E-01	5.58E-01
IL17A (pg/mL)	Immunology	4.76E-02	4.64E-01	6.64E-05	9.99E-01	3.78E-02	9.07E-01
Contrast-glcm	Radiomics	4.42E-02	4.83E-01	5.04E-02	5.15E-01	1.76E-02	9.07E-01
sCD40LG (pg/mL)	Immunology	4.19E-02	4.95E-01	1.02E-01	2.79E-01	4.97E-02	9.07E-01
Mean Corpuscular Hemoglobin (pg)	Clinical Pathology	4.08E-02	4.99E-01	4.25E-01	1.43E-03	3.03E-01	2.98E-02
Total Protein (g/dL)	Clinical Pathology	3.89E-02	5.04E-01	2.48E-01	4.54E-02	8.94E-02	7.93E-01
IL4 (pg/mL)	Immunology	3.89E-02	5.04E-01	4.18E-02	5.44E-01	3.63E-02	9.07E-01
IFNG (pg/mL)	Immunology	3.53E-02	5.28E-01	4.46E-02	5.33E-01	3.30E-02	9.07E-01
IL10 (pg/mL)	Immunology	3.34E-02	5.39E-01	4.02E-02	5.50E-01	2.77E-02	9.07E-01
Creatinine (mg/dL)	Clinical Pathology	3.15E-02	5.50E-01	4.17E-02	5.44E-01	1.61E-01	3.89E-01
IL2 (pg/mL)	Immunology	3.10E-02	5.50E-01	9.73E-02	2.82E-01	5.69E-02	9.07E-01
IL12B (pg/mL)	Immunology	2.89E-02	5.64E-01	4.06E-02	5.50E-01	2.87E-02	9.07E-01
Absolute Eosinophil (10^3/μL)	Clinical Pathology	2.57E-02	5.87E-01	9.72E-02	2.82E-01	8.54E-02	8.20E-01
Aspartate Aminotransferase (μL)	Clinical Pathology	2.55E-02	5.87E-01	1.81E-02	7.47E-01	8.46E-02	8.20E-01
Elongation-shape	Radiomics	1.87E-02	6.51E-01	2.20E-01	5.86E-02	1.78E-01	3.06E-01
Mean Corpuscular Volume (fL)	Clinical Pathology	1.88E-02	6.51E-01	2.76E-01	3.14E-02	1.28E-02	9.24E-01
IL13 (pg/mL)	Immunology	1.51E-02	6.92E-01	3.49E-02	5.98E-01	2.77E-02	9.07E-01
Hematocrit (%)	Clinical Pathology	1.30E-02	7.16E-01	1.05E-01	2.78E-01	7.53E-02	9.07E-01
IL1B (pg/mL)	Immunology	1.26E-02	7.16E-01	4.98E-02	5.15E-01	5.87E-02	9.07E-01
Gray Level Non-Uniformity Normalized-glszm	Radiomics	1.22E-02	7.16E-01	4.92E-02	5.17E-01	5.69E-02	9.07E-01
Alanine Aminotransferase (μL)	Clinical Pathology	9.38E-03	7.56E-01	8.16E-02	3.44E-01	1.18E-02	9.24E-01
Red Cell Distrubtion Width – Coefficient (%)	Clinical Pathology	8.80E-03	7.61E-01	2.54E-01	4.40E-02	2.98E-02	9.07E-01
Lactate Dehydrogenase (μL)	Clinical Pathology	8.46E-03	7.61E-01	6.67E-02	4.24E-01	1.79E-02	9.07E-01
Red Blood Cell (MM/μL)	Clinical Pathology	6.64E-03	7.90E-01	5.49E-02	4.88E-01	8.01E-02	8.71E-01
Difference Average-glcm	Radiomics	5.38E-03	8.12E-01	6.72E-02	4.24E-01	1.77E-02	9.07E-01
Hemoglobin (g/dL)	Clinical Pathology	2.51E-03	8.79E-01	1.50E-01	1.62E-01	1.54E-01	4.18E-01
IL5 (pg/mL)	Immunology	2.51E-03	8.79E-01	1.07E-02	8.44E-01	2.42E-03	9.79E-01

Red Cell Distrubtion Width – Std. Dev. (fL)	Clinical Pathology	1.75E-03	8.95E-01	4.16E-01	1.43E-03	3.20E-02	9.07E-01
CSF2 (pg/mL)	Immunology	1.72E-03	8.95E-01	7.82E-02	3.59E-01	4.09E-02	9.07E-01
TNF (pg/mL)	Immunology	1.48E-03	8.98E-01	3.40E-02	5.99E-01	5.06E-02	9.07E-01
Absolute Monocyte ($10^3/\mu L$)	Clinical Pathology	1.03E-03	9.08E-01	2.22E-01	5.86E-02	2.26E-01	1.20E-01
VEGFA (pg/mL)	Immunology	1.01E-03	9.08E-01	6.95E-02	4.16E-01	5.75E-03	9.57E-01
IDM-glcm	Radiomics	2.14E-05	9.87E-01	8.55E-02	3.31E-01	1.75E-02	9.07E-01
ID-glcm	Radiomics	1.51E-05	9.87E-01	8.69E-02	3.31E-01	1.77E-02	9.07E-01

p-values corrected for the false-discovery rate (p_{fdr}) were attained using the Benjamini–Hochberg correction on the 137 comparisons. ANOVA, analysis of variance; glszm, gray-level size zone matrix; ngtdm, neighboring gray-tone difference matrix; gldm, gray-level dependence matrix; glcm, gray-level co-occurrence matrix; glrlm, gray-level run length matrix; IDM, inverse difference moment; IDMN, inverse difference moment normalized; IL, interleukin; IDN, inverse difference normalized; IFN, interferon; MCP, monocyte chemoattractant protein 1; MCC, maximal correlation coefficient; IMC, informational measure of correlation; MIP, macrophage inflammatory protein; sCD40LG, soluble CD40 ligand; CSF2, colony stimulating factor 2; VEGFA, vascular endothelial growth factor A; ID, inverse difference; SARS-CoV-2, severe acute respiratory syndrome coronavirus 2. Immunological features follow HUGO Gene Nomenclature Committee (HGNC) nomenclature (Home | HUGO Gene Nomenclature Committee (genenames.org)).

Supplemental Table 3: Description of examined models.

Models; scikit-learn function	Description/Rationale	Key Parameters
Support Vector Machine; svm.SVC	Discriminative learning, handles non-linearity (for this kernel)	Kernel: radial basis function<br>Regularization: L2<br>Gamma: $1/(n_{feature} \times \sigma_X^2)$
Logistic Regression; linear_model. LogisticRegression	Discriminative learning, Linear	Solver: limited-memory Broyden–Fletcher–Goldfarb–Shanno (lmbfgs)<br>Regularization: L2
Decision Tree; tree. DecisionTreeClassifier	Discriminative learning, handles non-linearity, tree-based	No max depth of the tree<br>Minimum samples to split: 2<br>Minimum samples for leaf: 1
Random Forest; ensemble. RandomForestClassifier	Discriminative learning, handles non-linearity, tree-based, ensemble model	Number of trees: 100<br>No max depth of the trees<br>Max Features: 'sqrt'
K-Nearest Neighbors; neighbors. KNeighborsClassifier	Instance-based learning, handles non-linearity	Number of neighbors: 5<br>Weights: uniform<br>Metric: Euclidean distance
Gaussian Naïve Bayes; naive_bayes.GaussianNB	Generative learning, probabilistic kernel	Portion of the largest variance that is added to variances: 1e-9
Linear Discriminate Analysis; discriminant_analysis. LinearDiscriminantAnalysis	Generative learning, linear	Solver: singular value decomposition<br>No shrinkage
eXtreme Gradient Boosting; xgboost.sklearn. XGBClassifier	Gradient boosting learning, handles non-linearity, tree-based, ensemble model	Number of boosting rounds: 100<br>Learning rate (eta): 0.3<br>Max depth: 6

The examined models and key parameters are given as well as categorical descriptors. For all models, default parameters were used to simplify interpretation and comparison to other implementations of these models.

Supplemental Material 1: Full feature list.

The complete list of radiomic, clinical pathological, and immunological features used in this study is appended below. Features are grouped by domain and by inclusion/exclusion based on domain-specific feature screening and data-driven feature selection.

Radiomics Features

- Included
  - Voxel Volume – shape
  - Maximum 3D Diameter – shape
  - Mesh Volume – shape
  - Sphericity – shape
  - Compactness 1 – shape
  - Flatness – shape
  - Surface Area – shape
  - Maximum 2D Diameter Column – shape
  - Spherical Disproportion – shape
  - Joint Average – glcm
  - Joint Energy – glcm
  - Sum Average – glcm
  - Sum Entropy – glcm
  - Dependence Entropy – gldm
  - Gray Level Non-Uniformity – gldm
  - Small Dependence High Gray Level Emphasis – gldm
  - Interquartile Range – first order
  - Skewness – first order
  - Uniformity – first order
  - Median – first order
  - Root Mean Squared – first order
  - Entropy – first order
  - Mean – first order
  - Gray Level Non-Uniformity Normalized – glrlm
  - Gray Level Variance – glszm
  - Zone Variance – glszm
  - Size Zone Non-Uniformity – glszm
  - Gray Level Non-Uniformity – glszm
  - Large Area Emphasis – glszm
  - Small Area High Gray Level Emphasis – glszm
  - Contrast – ngtdm
  - Busyness – ngtdm
- Excluded by mRMR-permute feature selection
  - Compactness 2 – shape

- Major Axis Length – shape
- Least Axis Length – shape
- Elongation – shape
- Minor Axis Length – shape
- Maximum 2D Diameter Row – shape
- Autocorrelation – glcm
- Joint Entropy – glcm
- Cluster Shade – glcm
- Idmn – glcm
- Contrast – glcm
- Difference Variance – glcm
- Idn – glcm
- Idm – glcm
- Correlation – glcm
- MCC – glcm
- Sum Squares – glcm
- Cluster Prominence – glcm
- IMC 2 – glcm
- IMC 1 – glcm
- Difference Average – glcm
- Id – glcm
- Cluster Tendency – glcm
- Gray Level Variance – gldm
- High Gray Level Emphasis – gldm
- Dependence Non-Uniformity – gldm
- Large Dependence High Gray Level Emphasis – gldm
- Low Gray Level Emphasis – gldm
- Energy – first order
- Robust Mean Absolute Deviation – first order
- Mean Absolute Deviation – first order
- Standard Deviation – first order
- Total Energy – first order
- 90 Percentile – first order
- Range – first order
- Variance – first order
- 10 Percentile – first order
- Kurtosis – first order
- Maximum – first order
- Short Run Low Gray Level Emphasis – glrlm
- Gray Level Variance – glrlm

  - Low Gray Level Run Emphasis – glrlm
  - Gray Level Non-Uniformity – glrlm
  - Short Run High Gray Level Emphasis – glrlm
  - Run Length Non-Uniformity – glrlm
  - Long Run High Gray Level Emphasis – glrlm
  - Run Entropy – glrlm
  - High Gray Level Run Emphasis – glrlm
  - Gray Level Non-Uniformity Normalized – glszm
  - Size Zone Non-Uniformity Normalized – glszm
  - Large Area Low Gray Level Emphasis – glszm
  - Large Area High Gray Level Emphasis – glszm
  - High Gray Level Zone Emphasis – glszm
  - Small Area Emphasis – glszm
  - Zone Entropy – glszm
  - Coarseness – ngtdm
  - Complexity – ngtdm
  - Strength – ngtdm
- Excluded by domain-specific feature screening
  - Surface Volume Ratio – shape
  - Maximum 2D Diameter Slice – shape
  - Maximum Probability – glcm
  - Difference Entropy – glcm
  - Inverse Variance – glcm
  - Small Dependence Emphasis – gldm
  - Dependence Non-Uniformity Normalized – gldm
  - Large Dependence Emphasis – gldm
  - Large Dependence Low Gray Level Emphasis – gldm
  - Dependence Variance – gldm
  - Small Dependence Low Gray Level Emphasis – gldm
  - Minimum – first order
  - Run Variance – glrlm
  - Long Run Emphasis – glrlm
  - Short Run Emphasis – glrlm
  - Run Percentage – glrlm
  - Long Run Low Gray Level Emphasis – glrlm
  - Run Length Non-Uniformity Normalized – glrlm
  - Zone Percentage – glszm
  - Low Gray Level Zone Emphasis – glszm
  - Small Area Low Gray Level Emphasis – glszm

Clinical Pathology Features

- Included
  - Absolute basophil count (10^3/μL)
  - Absolute reticulocyte count (10^3/μL)
  - Blood urea nitrogen concentration (mg/dL)
- Excluded by mRMR-permute feature selection
  - Absolute neutrophil count (10^3/μL)
  - Absolute eosinophil count (10^3/μL)
  - Absolute lymphocyte count (10^3/μL)
  - Absolute monocyte count (10^3/μL)
  - Alanine aminotransferase (μL)
  - Albumin concentration (g/dL)
  - Albumin–globulin ratio
  - Aspartate aminotransferase (μL)
  - Creatinine (mg/dL)
  - Hematocrit (%)
  - Hemoglobin (g/dL)
  - Lactate dehydrogenase (μL)
  - Mean corpuscular hemoglobin (pg)
  - Mean corpuscular hemoglobin concentration (g/L)
  - Mean corpuscular volume (fL)
  - Platelet count (10^3/μL)
  - Platelet crit (%)
  - Red blood cell count (MM/μL)
  - Red cell distribution width – coefficient of variation (%)
  - Red cell distribution width – standard deviation (fL)
  - Total protein concentration (g/dL)
  - White blood cell count (10^3/μL)
- Excluded by domain-specific feature screening
  - Percent basophil (%)
  - Percent eosinophil (%)
  - Percent lymphocyte (%)
  - Percent monocyte (%)
  - Percent neutrophil (%)
  - Percent reticulocyte (%)
  - Mean platelet volume (fL)
  - Platelet distribution width (fL)
  - Platelet large cell – ratio (%)
  - Globulin (g/dL)
  - Hemolysis (0-4)

- Lipemia (0-4)

Immunology Features

- Included [none]
- Excluded by mRMR-permute feature selection
  - CSF2 (pg/mL)
  - IFNA1 (pg/mL)
  - IFNG (pg/mL)
  - IL10 (pg/mL)
  - IL12B (pg/mL)
  - IL13 (pg/mL)
  - IL15 (pg/mL)
  - IL17A (pg/mL)
  - IL18 (pg/mL)
  - IL1B (pg/mL)
  - IL6R (pg/mL)
  - IL2 (pg/mL)
  - IL4 (pg/mL)
  - IL5 (pg/mL)
  - IL6 (pg/mL)
  - CXCL8 (pg/mL)
  - CCL2 (pg/mL)
  - CCL3 (pg/mL)
  - CCL4 (pg/mL)
  - TNF (pg/mL)
  - VEGFA (pg/mL)
  - sCD40LG (pg/mL)
  - Excluded by domain-specific feature screening [none]

mRMR, minimum redundancy maximum relevance; glszm, gray-level size zone matrix; ngtdm, neighboring gray-tone difference matrix; gldm, gray-level dependence matrix; glcm, gray-level co-occurrence matrix; glrlm, gray-level run length matrix; IDM, inverse difference moment; IDMN, inverse difference moment normalized; IL, interleukin; IDN, inverse difference normalized; IFN, interferon; MCP, monocyte chemoattractant protein 1; MCC, maximal correlation coefficient; IMC, informational measure of correlation; MIP, macrophage inflammatory protein; sCD40LG, soluble CD40 ligand; CSF2, granulocyte-macrophage colony-stimulating factor; VEGFA, vascular endothelial growth factor A; ID, inverse difference. Immunological feature names follow HUGO Gene Nomenclature Committee (HGNC) nomenclature (Home | HUGO Gene Nomenclature Committee (genenames.org)).

Supplemental Figure 1: Change-from-baseline violin plots for mRMR-permute features.

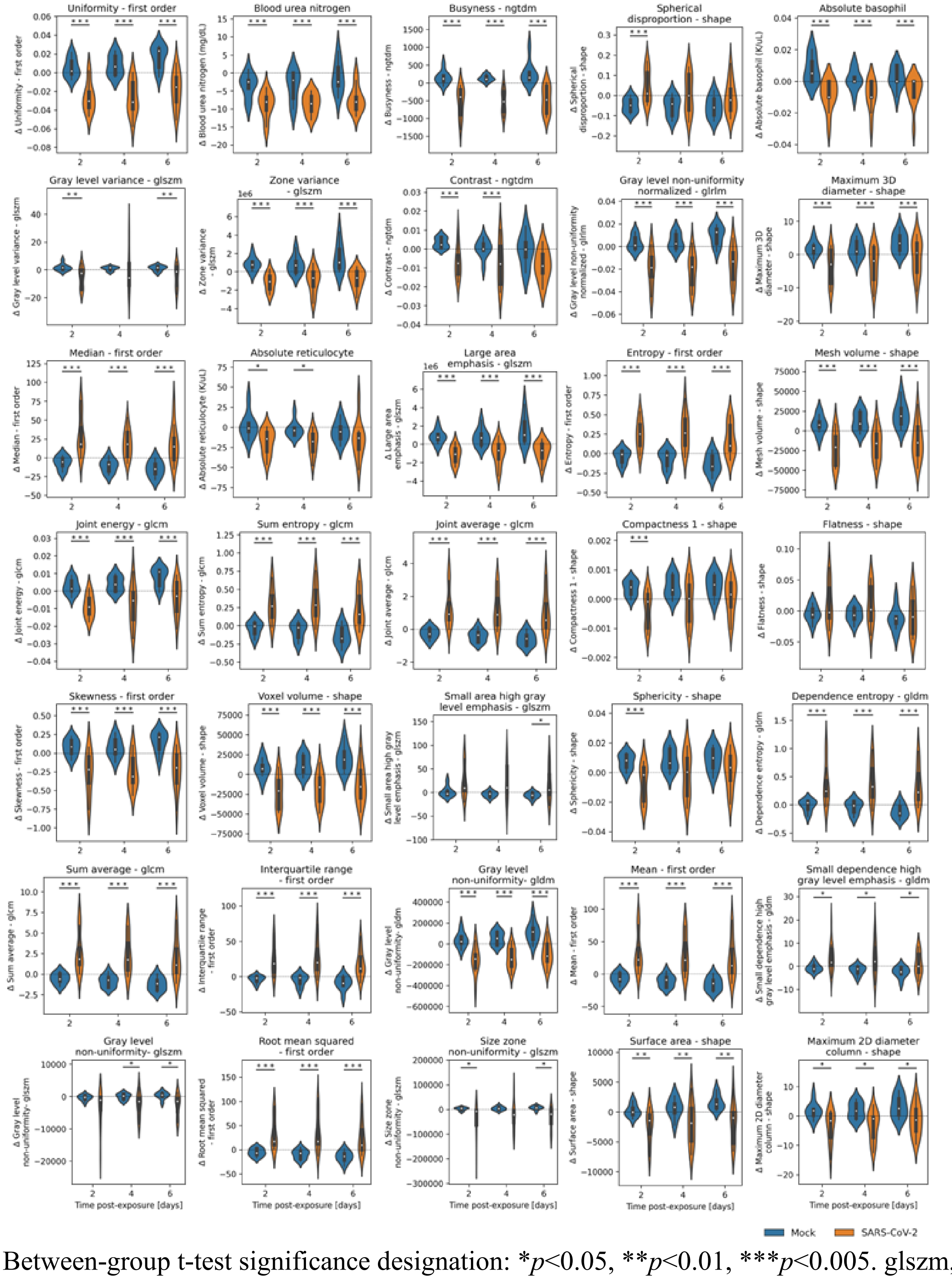

Between-group t-test significance designation: *p<0.05, **p<0.01, ***p<0.005. glszm, gray-level size zone matrix; gldm, gray-level dependence matrix; glcm, gray-level co-occurrence matrix; SARS-CoV-2, severe acute respiratory syndrome coronavirus 2.
